# Hyperuricemia contributes to glucose intolerance of hepatic inflammatory macrophages and impairs the insulin signaling pathway *via* IRS2-proteasome degradation

**DOI:** 10.3389/fimmu.2022.931087

**Published:** 2022-09-13

**Authors:** Hairong Zhao, Jiaming Lu, Furong He, Mei Wang, Yunbo Yan, Binyang Chen, De Xie, Chenxi Xu, Qiang Wang, Weidong Liu, Wei Yu, Yuemei Xi, Linqian Yu, Tetsuya Yamamoto, Hidenori Koyama, Wei Wang, Chenggui Zhang, Jidong Cheng

**Affiliations:** ^1^Department of Endocrinology, Xiang’an Hospital of Xiamen University, Xiamen University, Xiamen, China; ^2^Yunnan Provincial Key Laboratory of Entomological Biopharmaceutical Research and Development (R&D), College of Pharmacy, Dali University, Dali, China; ^3^Department of Diabetes, Endocrinology and Clinical Immunology, Hyogo College of Medicine, Nishinomiya, Japan

**Keywords:** hyperuricemia, insulin resistance of macrophage, insulin signaling, IRS2-proteasome degradation, inflammatory macrophage

## Abstract

**Aim:**

Numerous reports have demonstrated the key importance of macrophage-elicited metabolic inflammation in insulin resistance (IR). Our previous studies confirmed that hyperuricemia or high uric acid (HUA) treatment induced an IR state in several peripheral tissues to promote the development of type 2 diabetes mellitus (T2DM). However, the effect of HUA on glucose uptake and the insulin sensitivity of macrophages and its mechanism is unclear.

**Methods:**

To assess systemic IR, we generated hyperuricemic mice by urate oxidase knockout (UOX-KO). Then, glucose/insulin tolerance, the tissue uptake of 18F-fluorodeoxyglucose, body composition, and energy balance were assessed. Glucose uptake of circulating infiltrated macrophages in the liver was evaluated by glucose transporter type 4 (GLUT-4) staining. Insulin sensitivity and the insulin signaling pathway of macrophages were demonstrated using the 2-NBDG kit, immunoblotting, and immunofluorescence assays. The immunoprecipitation assay and LC-MS analysis were used to determine insulin receptor substrate 2 (IRS2) levels and its interacting protein enrichment under HUA conditions.

**Results:**

Compared to WT mice (10 weeks old), serum uric acid levels were higher in UOX-KO mice (WT, 182.3 ± 5.091 μM versus KO, 421.9 ± 45.47 μM). Hyperuricemic mice with metabolic disorders and systemic IR showed inflammatory macrophage recruitment and increased levels of circulating proinflammatory cytokines. HUA inhibited the nuclear translocation of GLUT-4 in hepatic macrophages, restrained insulin-induced glucose uptake and glucose tolerance, and blocked insulin IRS2/PI3K/AKT signaling. Meanwhile, HUA mediated the IRS2 protein degradation pathway and activated AMPK/mTOR in macrophages. LC-MS analysis showed that ubiquitination degradation could be involved in IRS2 and its interacting proteins to contribute to IR under HUA conditions.

**Conclusion:**

The data suggest that HUA-induced glucose intolerance in hepatic macrophages contributed to insulin resistance and impaired the insulin signaling pathway *via* IRS2-proteasome degradation

## Introduction

Hyperuricemia is customarily defined as a serum uric acid (UA) level of >420 μM in humans, predisposing them to gout by the formation of urate crystals (1). In humans and great apes, UA is the end-product of purine degradation whereas, in other mammals, it is further degraded into allantoin, a more soluble metabolic product by urate oxidase (UOX) (2), an enzyme generally located in the liver. Accumulating evidence from clinical observational studies suggests the involvement of asymptomatic hyperuricemia, independent of crystal formation, in hypertension, atherosclerosis, acute and chronic kidney disease, obesity, metabolic syndrome, fatty liver, insulin resistance (IR), and diabetes (3–5). Our previous studies also confirmed that hyperuricemia induced an IR state in several peripheral tissues (6–8), including the liver, myocardium, skeletal muscles, and adipose tissue as well as pancreatic β cells (9), and was a strong risk factor for type 2 diabetes mellitus (T2DM).

T2DM has both an inflammatory and metabolic etiology. Concerning glucose metabolism, insulin mainly acts on the liver, muscles, and adipose tissue, and the responses of these targets to insulin and other hormones determine the circulating concentrations of glucose, fatty acids, and other metabolites (10, 11). In contrast, chronic low-grade inflammation and immune system activation are implicated in the pathological process of local and systemic IR and T2DM (12–14). Numerous studies have demonstrated the key importance of macrophage-elicited metabolic inflammation in IR (15, 16). In obesity and T2DM, macrophages infiltrate metabolic organs such as the liver and adipose tissue, leading to low-grade inflammation that impairs insulin action (17–19).

Liver-resident macrophages, also called Kupffer cells (KCs), represent 80–90% of the whole-body macrophage population and are characterized by the expression of canonical macrophage markers, including F4/80, CD14, CD68, and CD11b (20, 21). KCs are activated in obesity to a more inflammatory or M1 state (22), resulting in the migration of inflammatory monocytes (Ly6C+ in mice) into the liver, where they differentiate into monocyte-derived macrophages, exacerbating the obesity-induced hepatic inflammation (23, 24). Thus, the liver undergoes a spectrum of changes ranging from benign steatosis to fibrosis and cirrhosis (25).

Hyperuricemia is also associated with chronic low-grade tissue inflammation, manifested as elevated serum chemokine ligand 2 levels, increased monocyte recruitment (26), and chronic inflammatory cell infiltration into the kidney (26). Clinical studies and our experiments have found associations between serum UA levels and the development of IR (27, 28); however, whether macrophages infiltrate the liver and lead to intrinsic IR and the underlying mechanisms of the process remain unknown. In the current study, we constructed a mouse model of UOX knockout (UOX-KO) to investigate the role of high UA (HUA) in hepatic macrophage recruitment and IR. Additionally, we explored the effect of UA on the insulin signaling pathway in macrophages from mice.

## Materials and methods

### Animals and treatments

UOX-KO C57BL/6J mice were generated as described previously (29). Adult male wild-type (WT) C57BL/6J mice weighing 22–25 g were obtained from the Xiamen University Laboratory Animal Center. All mice were housed in a specific-pathogen-free facility under 12-h light/dark cycles in a temperature-controlled environment (22–25°C) with 40–70% humidity and free access to food and water. All experimental procedures and animal housing in this study were designed and conducted in accordance with the approval of the Institutional Animal Care and Use Committee of Xiamen University, China (Animal Ethics number: XMULAC20200122).

**Figure 1:**
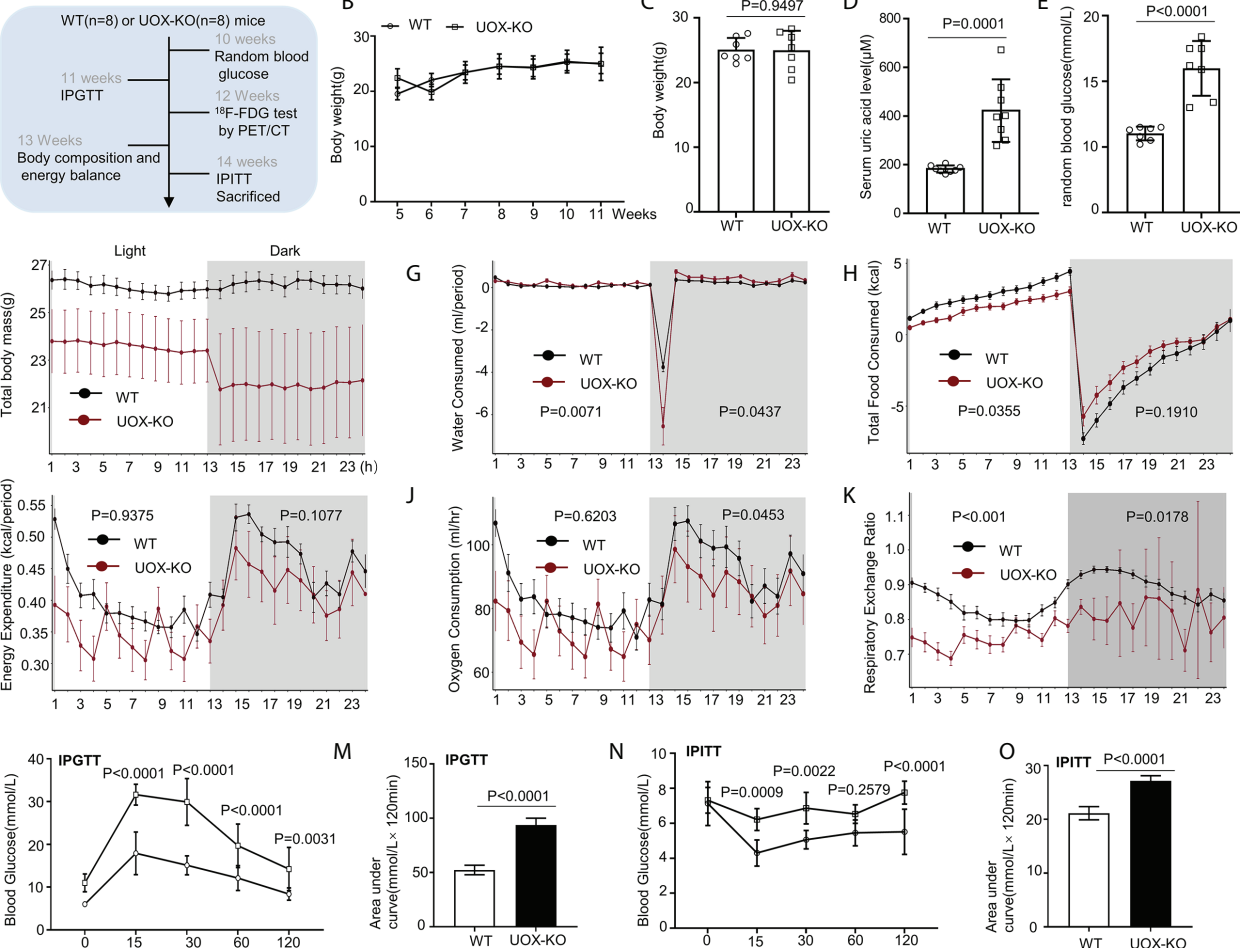
Body composition, energy homeostasis, and glucose metabolism in male wild-type (WT) and urate oxidase-knockout (UOX-KO) mice. **(A)** Random blood glucose measurements, intraperitoneal glucose tolerance tests (IPGTTs), ^18^F-FDG tests, and intraperitoneal insulin tolerance tests (IPITTs) were carried out according to the relevant timelines. **(B)** The body weights of WT and UOX-KO mice were continuously monitored for six weeks, and the body weights of 15-week-old mice were also recorded **(C)**. **(D)** Serum uric acid levels. **(E)** Random blood glucose levels. **(F–J)** Water and food consumed, energy expenditure, and oxygen consumption relative to the body weight of the mice. **(K)** Respiratory exchange ratio (CO_2_ exhaled/O_2_ inhaled). **(L)** IPGTT blood glucose values in 11-week-old mice and area under the receiver operating characteristic curve (AUC) for the IPGTT **(M)**. **(N)** IPITT blood glucose values in 14-week-old mice and AUC for ITT **(O)**. All the mice used in the experiment were male. Data are the mean ± SD (n = 8). Statistics: Two-way repeated-measures ANOVA **(B, F–K, L, N)**; Student’s t-test **(C–E, M, O)**. Differences were considered significant at P < 0.05.

The mice were assigned to two experimental groups: the WT group (n = 8) and the UOX-KO group (n = 8). A schematic diagram of the timeline for animal treatment and the study design is shown in **Figure 1A**. At the end of the experiment (14 weeks), the mice were fasted all night and then anesthetized with 1.5% isoflurane in a mixture of 30% O_2_ and 69% N_2_O. Blood samples were collected from the sinus orbital vein (200 μL) to evaluate the release of proinflammatory cytokines. The mice were euthanized by cervical dislocation (WT = 6, KO = 6). Serum was separated by centrifugation at 3,000 rpm for 15 min at 4°C. The liver was collected to assess the infiltration and activation of macrophages.

To isolate macrophages from the liver, WT (n = 5) and KO (n = 5) mice were anesthetized with 1.5% isoflurane in a mixture of 30% O_2_ and 69% N_2_O. The abdomen was opened, and the hepatic portal vein was dissociated. Hepatic perfusion was successively performed for 2 min using Hank’s balanced salt solution and collagenase IV (37°C) (Sigma). Subsequently, the liver was removed, and macrophages were separated using the following methods. The number of mice (n) used for each endpoint is given in each figure or figure legend.

### Glucose and insulin tolerance tests

Body weight and food intake were recorded twice a week in mice housed individually (except for the survival study). In detail, to analyze serum uric acid (at 10 weeks) and random blood glucose (at 10 weeks) levels, and for the intraperitoneal glucose tolerance test (IPGTT) (at 11 weeks) and intraperitoneal insulin tolerance test (IPITT) (at 14 weeks), blood samples were collected in the morning from the tail vein without anesthesia after fasting overnight. The serum uric acid and random blood glucose levels in WT mice and UOX-KO mice were assessed using a blood glucose and uric acid meter (EA-11, Sinocare). IPGTT and IPITT were performed in mice that were fasted for 8 or 4 h. For the IPGTT, 11-week-old mice were fasted overnight before the intraperitoneal administration of glucose at 2 g/kg body weight. In another set of experiments, we performed IPITT in 14-week-old mice. Normal WT mice and UOX-KO mice that were fasted for 4 h were intraperitoneally injected with insulin at 0.5 units/kg body weight. Tail blood glucose concentrations were monitored at 0, 15, 30, 60, and 120 min using a glucometer. Incremental areas under the curve (AUC) were calculated from 0 to 120 min for glucose and insulin.

### Body composition and energy balance

Body weight and food intake were monitored daily to obtain body weight gain and gross energy intake. The evaluation of energy homeostasis and metabolic parameters at the 13-week interval in WT and UOX-KO mice was continuously conducted for three days using a Promethion Comprehensive High-resolution Behavioral Analysis System (Sable Promethion, Sable Systems International, USA). The experiment was conducted at optimum temperature (22–25°C) with a 12 h/12 h light-dark cycle. Metabolizable energy (ME) intake was calculated by subtracting the energy measured in feces and urine from the gross energy intake, determined by the daily food consumption and gross energy density. Energy efficiency was calculated as the percentage of body energy retained per ME intake, and energy expenditure was the difference between ME intake and energy gain.

### Tissue uptake of ^18^F-Fluorodeoxyglucose

^18^F-Fluorodeoxyglucose (FDG) (radiochemical purity >95%) was synthesized by the nucleophilic substitution method using an FDG-synthesizing instrument (Center for Molecular Imaging and Translational Medicine, School of Public Health, Xiamen University, Xiamen, China). The mice that were fasted for 16 h were restrained, injected with insulin (0.75 units/kg) diluted in 0.9% saline for 5 min, and then intravenously injected with ^18^F-FDG (200–300 µCi/mouse). The mice were deeply anesthetized with 1.5% isoflurane in a mixture of 30% O_2_ and 69% N_2_O. Then, they underwent small-animal positron emission tomography (PET) and microcomputed tomography. Whole-body PET images were acquired 30 min later using an acquisition time of 30 min (window width: 20%; matrix: 256 × 256; medium zoom). The PET/CT images were analyzed using InVivoScope software (Bioscan Inc.). To quantify the radioactive signals in tissues, regions of interest (ROIs) were drawn and counted on the PET/CT images of the brain, kidney, liver, heart, fat, and muscle, and their location was confirmed by CT. The differential uptake ratio (DUR) was used as an index of radiotracer uptake in tissues and calculated as DUR = (tissue counts [cpm] per g of tissue)/(injected dose counts per g of body weight). The results are expressed as a percentage of the injected dose per gram (%ID/g).

### Serum inflammatory factors

Antibodies against serum interleukin 1β (IL-1β, Cat# 88-5019-88, RRID: AB_2574807), tumor necrosis factor-alpha (TNF-α, Cat# 88-7324-22, RRID:A B_2575076), IL-6 (Cat# 88-7064-22, RRID: AB_2574986), and monocyte chemoattractant protein 1 (MCP-1, Cat# 88-7391-88, RRID: AB_2575113) were purchased from Thermo Fisher Scientific and measured using commercially available cytokine enzyme-linked immunosorbent assay (ELISA) kits.

### Transmission electron microscopy

Liver samples were isolated from WT and UOX-KO mice and fixed in 2% glutaraldehyde and 4% paraformaldehyde in 0.1-M sodium cacodylate (pH=7.4), treated with 10% gelatin solution in sodium cacodylate buffer, and incubated with 2% osmium tetroxide. Sections measuring 40 nm were cut using a Leica EM TP ultramicrotome (Leica Microsystems) and placed within grids stained with a 1:1 mixture of 3% uranyl acetate and 50% acetone for 30–60 s. The grids were imaged with a JEM 1400 transmission electron microscope (JEOL) at ×1,200 for low magnification and ×12,000 for high magnification (unless otherwise noted) using Gatan Microscopy Suite software (Gatan). The cells were identified as described previously (30).

### Isolation of hepatic macrophages

Livers were collected, digested with collagenase IV (Sigma) and DNase, and filtered through a 70-μM cell strainer (Biosharp). Single-cell suspensions were prepared after removing red blood cells using ammonium-chloride-potassium (ACK) lysis buffer (Sigma). Hepatic leukocytes were isolated by 30%, 37%, and 70% Percoll (GE Healthcare) density gradient centrifugation. Cell counts were determined on a Coulter counter (Thermo Fisher). The cells were washed with phosphate-buffered saline (PBS) solution three times and stained with fluorochrome-conjugated monoclonal antibodies, which included fluorescein (FITC)-conjugated rat anti-mouse CD45 (1:100, Cat# 561886, RRID: AB_395576, BD Biosciences) and allophycocyanin (APC)-conjugated CD11b (1:100, Cat# 553312, RRID: AB_398535, BD Biosciences). The samples were analyzed by flow cytometry (CytoFLEX S). The analysis was performed with FlowJo software (Tree Star Inc., San Carlos, CA, USA). Total macrophages were identified as CD45^+^CD11b^+^.

### Cell isolation, culture, and treatment

The established THP-1 cell line (CLS Cat# 300356/p804_THP-1, RRID: CVCL_0006) was obtained from the Chinese Academy of Science (ATCC, Shanghai). THP-1 cells were cultured and differentiated into macrophages as described previously (31). To assess IR in THP-1 cells, the cells were sub-cultured in 6-cm culture dishes, exposed to 15 mg/dL of UA for 24 h, harvested by scraping, and stored at -80°C.

### Peritoneal macrophages

After the intraperitoneal injection of 3% Brewer thioglycolate medium (BD Biosciences, Franklin Lakes, NJ, USA) for 4 days, peritoneal macrophages were isolated from the peritoneal cavity of male UOX-KO and WT mice. Briefly, the mouse peritoneal cavity was rinsed with 5 mL of ice-cold PBS (pH=7.4) containing 2% fetal bovine serum (FBS). The peritoneal fluid was collected, and the cell concentration was adjusted to 3×10^6^ nucleated cells/mL in Dulbecco’s modified Eagle medium (DMEM) supplemented with 10% FBS. The macrophages were cultured in a humidified incubator at 37°C for 2 h. Non-adherent cells were removed by gentle washing with warm PBS three times. To evaluate the effect of UA on insulin signaling pathways, the cells were sub-cultured in 6-well plates (2.5 × 10^5^ cells/well) and exposed to UA (0, 5, 10, and 15 mg/dL) for 24 h or 100-nM insulin for 0.5 h.

### Bone-marrow-derived

macrophages Bone-marrow cells were isolated from the femur and tibia of male WT mice. The marrow was flushed from the bones with DMEM loaded into a 1-mL syringe. Bone marrow cells were cultured at a concentration of 3×10^6^ cells/mL of DMEM medium containing 10% FBS and 20 ng/mL of granulocyte-colony-stimulating factor (G-CSF) (#576406, Biolegend) to differentiate them into BMDMs. On day 7, adherent cells became mature macrophages. On day 8, the differentiated BMDMs were re-plated with DMEM without G-CSF overnight and then stimulated with 15 mg/mL of UA for 24 h.

Primary peritoneal macrophages and BMDMs were identified as CD45^+^F4/80^+^.

### Quantitative immunoblotting

Quantitative immunoblotting was accomplished as we described previously (9). A high dilution of β-actin rabbit mAb (1:10000, ABclonal Cat# AC026, RRID: AB_2768234, China) was used as an internal loading control. The imaging of blots was performed using an Azure C300 Digital Imager (Azure Biosystems) after incubation with secondary antibodies (1:1000, G-21234, RRID: AB_1500696, Invitrogen, USA). The analysis was carried out in the Image Lab, and the optical density of the bands was quantified using ImageJ (National Institutes of Health, Bethesda, MD, USA) and normalized to the loading controls, as applicable. The antibodies and their concentrations were as follows (antibody, dilution, catalog number): insulin receptor substrate 1 (IRS1; 1:1000, #2382, RRID: AB_330333), IRS2 (1:1000, Cat# 3089, RRID: AB_2125771), AS160 (C69A7) rabbit mAb (1:1000, Cat# 2670, RRID: AB_2199375), phospho-AS160 (Thr642) antibody (1:1000, Cat# 4228, RRID: AB_659940), PI3 kinase p85 (1:1000, Cat# 4292, RRID: AB_329869), PI3K p85 (Tyr458)/p55 (Tyr199) (1:1000, Cat# 4228, RRID: AB_659940), AKT (1:1000, Cat# 9272, RRID: AB_329827), phospho-AKT (Ser473) (D9E) (1:1000, Cat# 4060, RRID: AB_2315049), AMPKα (D5A2) rabbit mAb (1:1000, Cat# 5831, RRID: AB_10622186), phospho-AMPKα (Thr172) (D4D6D) rabbit mAb (1:500, Cat# 50081, RRID: AB_2799368), mTOR (1:1000, Cat# 2983, RRID: AB_2105622), phosphorylated mTOR (Ser2448), and rabbit mAb (1:1000, Cat# 2971, RRID: AB_330970). All the antibodies were purchased from Cell Signaling Technology.

### Cycloheximide chase assay

To evaluate whether HUA destabilized the IRS2 protein, THP-1 cells were treated with 15 mg/dL of HUA for 24 h. Subsequently, THP-1 cells were treated with 5-μM cycloheximide (CHX) (MedChemExpress) for 3 h.

### Immunofluorescence staining

Liver sections of UOX-KO mice underwent immunofluorescence staining as described previously (9). The antibodies used and their concentrations are listed below (antibody, dilution, catalog number, company): anti-glucose transporter 4 (anti-GLUT4, 1:400, Cat# ab654, RRID: AB_305554, Abcam, USA), LAMP1 (1:200, Cat# ab24170, RRID: AB_775978, Abcam), and anti-F4/80 macrophage marker (CI:A3-1, 1:100, Cat# ab6640, RRID: AB_1140040, Abcam). After primary antibody incubation, the sections were washed with PBS four times for 10 min at room temperature and incubated with a secondary antibody (1:1000, A32728 (RRID: AB_2633277) or A32742 (RRID : AB_2762825) or A32731 (RRID : AB_ 2633280) goat anti-rabbit/rat/mouse IgG [H+L] cross-adsorbed secondary antibody (Thermo Scientific, USA) for 1 h. For the negative control, the primary antibody was omitted during immunostaining. Cell nuclei were stained using a DAPI kit for 5 min. Images were acquired by confocal microscopy (Zeiss LSM 880 or FV1000 MPE-B, Olympus) and processed using ImageJ.

### Quantitative polymerase chain reaction with reverse transcription analysis

BMDMs were treated with HUA for 24 h. Total cellular RNA was extracted using a SteadyPure Universal RNA Extraction Kit II (AG21022, Accurate Biology, China), and cDNA was synthesized using a PrimeScript RT Master Mix (Perfect Real Time) kit (RR036A, Takara, Japan). The resulting cDNA was analyzed by qRT-PCR with reverse transcription using SYBR Green PCR Master Mix (Applied Biosystems). The samples were normalized to the β-actin housekeeping. Quantitative analysis was performed by the ^ΔΔ^CT method. The primers (5′–3′) used were designed on Primer-Blast and are shown in **Table 1**.

**Table 1:** Primer sequence.

Gene	ID	Forward Sequence	Reverse Sequence
Irs1	3667	CTGCACAACCGTGCTAAGG	CGTCACCGTAGCTCAAGTCC
Irs2	8660	CCTCACCCTGTAGTGCCTTC	AAGTCGATGTTGATGTACTCGC
Insr	3643	AAAACGAGGCCCGAAGATTTC	GAGCCCATAGACCCGGAAG
Marcks	4082	AGCCCGGTAGAGAAGGAGG	AGCCCGGTAGAGAAGGAGG
Syt7	9066	ACTCCATCATCGTGAACATCATC	TCGAAGGCGAAGGACTCATTG
Gclc	2729	GGCACAAGGACGTTCTCAAGT	CAGACAGGACCAACCGGAC
Stat1	6772	CAGCTTGACTCAAAATTCCTGGA	TGAAGATTACGCTTGCTTTTCCT
Crim1	51232	CCCTGTGACGAGTCCAAGTG	GGTTCCGTAAATCCCGAAGGT
Prkcd	5580	AACCATGAGTTTATCGCCACC	AGCGTTACATTGCCTGCATTT
il1rn	3557	CATTGAGCCTCATGCTCTGTT	CGCTGTCTGAGCGGATGAA
Map4k4	9448	GGAACACACTCAAAGAAGACTGG	GTGCCTATGAACGTATTTCTCCG
Anxa1	301	CTAAGCGAAACAATGCACAGC	CCTCCTCAAGGTGACCTGTAA

### Glucose uptake measurement by 2-NBDG

The glucose uptake of BMDMs was assessed by the fluorescent glucose analog, 2-NBDG. Briefly, the cells were treated with low-glucose FBS-free DMEM for 24 h. Then the medium was replaced with Krebs-Ringer-Bicarbonate (KRB) buffer containing insulin (final concentration, 100 nM) and 2-NBDG (final concentration, 100 μM) for 30 min at 37°C and analyzed by fluorometry at excitation and emission wavelengths of 485 and 535 nm, respectively.

### Immunoprecipitation assay

HEK-293T cells (Cat# BFN60810479) were obtained from the American Type Culture Collection. HEK-293T cells expressing Flag-IRS2 (HG17635-CF, Sino Biological Inc.) or the PCDH vector were grown on 15-cm plates until 90% confluency and then analyzed by immunoprecipitation (IP). After transfection for 2 days, the cells were lysed in lysis buffer (1% sodium deoxycholate, 1% Triton X-100, 150 mM NaCl, 1 mM EDTA, 50 mM Tris-HCl [pH=7.4]) containing a protease inhibitor cocktail (#HY-K0010, MCE). Pre-washed Flag-beads (#M8823, Millipore) were added. After immunoprecipitation for 18 h, Flag-beads were washed five times for 10 min each with lysis buffer containing protease/phosphatase inhibitor. Flag-tagged proteins were eluted. A loading buffer of 4× sodium dodecyl sulfate (SDS) was added to the supernatant after enrichment and the sample was boiled in a metal bath for 10 min. Then, the sample was subjected to SDS-polyacrylamide gel electrophoresis (PAGE) (8% separating gel) and western blotting analysis.

### LC-MS analysis and database search

Each IP assay was performed in triplicate. HEK-293T cells overexpressing Flag-IRS2 were grown on 15-cm plates until 90–95% confluency. The group set was as follows: IRS2 and UA+IRS2. After transfection for two days, the UA+IRS2 group was treated with 15 mg/dL of UA for 24 h, and then the cells were subjected to IP as mentioned above. LC-MS analysis was executed according to our previously described method (32). The peptides were desalted by StageTips (33, 34). Gene ontology (GO) and Kyoto Encyclopedia of Genes and Genomes (KEGG) analysis were conducted by DAVID tools (https://david.ncifcrf.gov/). A P-value of <0.01 was considered statistically significant.

### Statistical analysis

Statistical analysis was conducted using GraphPad Prism 8. Data from repeated cell experiments were presented as the mean ± SD (n ≥ 4). Bar graphs were also used to show individual values. If the sample was normally distributed, statistical analysis was conducted between multiple groups using one-way analysis of variance (ANOVA), followed by the Dunnett’s test or Student’s t-test. Differences were considered significant at a P-value of <0.05.

## Results

### UOX-KO mice exhibited metabolic disorders and systemic IR

Our previous studies demonstrated that UOX-KO mice spontaneously developed hyperuricemia and aberrant lipid metabolism, concomitant with abnormal hepatic fat accumulation (29). We have also confirmed that UOX-KO mice had hepatic steatosis (**Supplementary Figure 1**). Then, we evaluated body weight and glucose metabolism in the UOX-KO mice. Random blood glucose levels, IPGTT, ^18^F-FDG test, and IPITT were determined according to the timeline in **Figure 1A**. Systemic UOX-KO had no significant effect on body weight (**Figures 1B, C**, **F**), but the serum uric acid and random blood glucose levels were elevated compared to the WT mice (**Figures 1D, E**). To assess whole-body metabolic states and the mechanisms underlying stable weight and elevated blood glucose, we used indirect calorimetry in a Promethion Comprehensive High-resolution Behavioral Analysis System over three days in 13-week-old mice on a chow diet. Notably, IR resulted in decreased energy production by glucose utilization, contributing to the pathogenesis of T2DM. UOX-KO mice showed increased water consumption under both light and dark conditions (**Figure 1G**), whereas total food consumption increased and energy expenditure decreased under light conditions (**Figures 1H, I**). Additionally, oxygen consumption and the respiratory exchange ratio increased under dark conditions (**Figures 1J, K**), indicating decreased glycolysis and fat oxidation, respectively, under dark conditions (**Supplementary Data set 1**, https://figshare.com/s/90b6af1ad966dff6f244).

**Figure 2:**
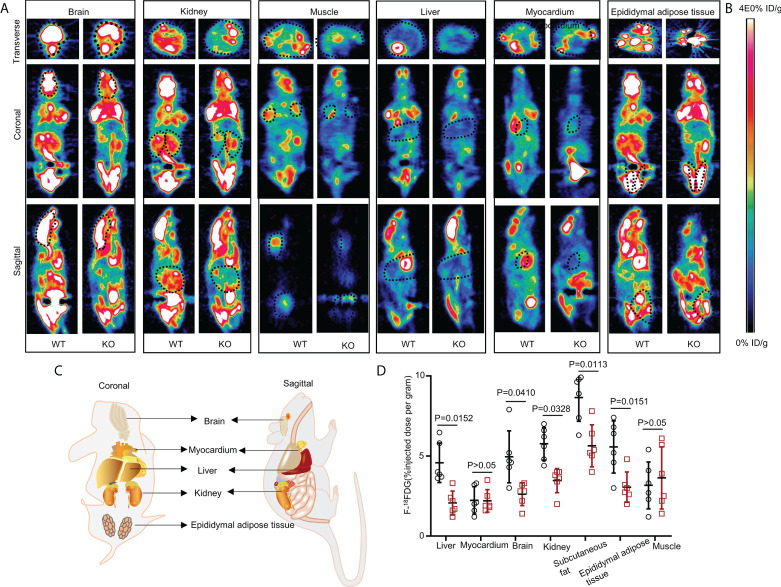
Whole-body ^18^FDG uptake in WT and UOX-KO mice. **(A)** Representative PET image (whole-body ^18^FDG uptake) of the brain, kidney, muscles, liver, myocardium, and epididymal adipose tissue in WT and UOX-KO mice. **(B)** The change in color represents the level of glucose uptake. **(C)** Schematic diagram of coronal and sagittal mice. **(D)** Quantitative measurement of ^18^FDG uptake in the brain, kidney, muscles, liver, myocardium, and epididymal adipose tissue (the value of the region of interest [ROI]; the dotted line circle). All the mice used in the experiment were male. Data are the mean ± SD (n = 6). Statistics: Tukey multiple comparisons test. Differences were considered significant at P < 0.05.

The UOX-KO mice exhibited glucose intolerance and insulin insensitivity compared to the WT mice, as assessed by GTT or ITT (**Figures 1L–O**). A pyruvate tolerance test (PTT) was performed to evaluate potential differences in liver-specific insulin sensitivity between the two genotypes of mice. Our preliminary results showed that the UOX-KO mice exhibited PTT compared to the WT mice (**Supplementary Figure 2**). Then, we reproduced this observation in male WT and UOX-KO mice using PET/CT imaging to assess ^18^F-FDG uptake. ^18^F-FDG uptake was lower in UOX-KO than in WT mice (**Figures 2A–D**). Glucose uptake was not seen in the liver, brain, kidney, and adipose tissue of the UOX-KO mice, suggesting that UOX-KO generates a systemic disorder of glucose uptake. There was no difference in glucose uptake by the myocardium and muscle between the two groups. Consistent with our previous observation in hyperuricemic mice, insulin-stimulated glucose uptake and metabolic homeostasis were impaired.

### UOX-KO mice liver recruited inflammatory macrophages and exhibited increased circulating proinflammatory cytokines

**Figure 3:**
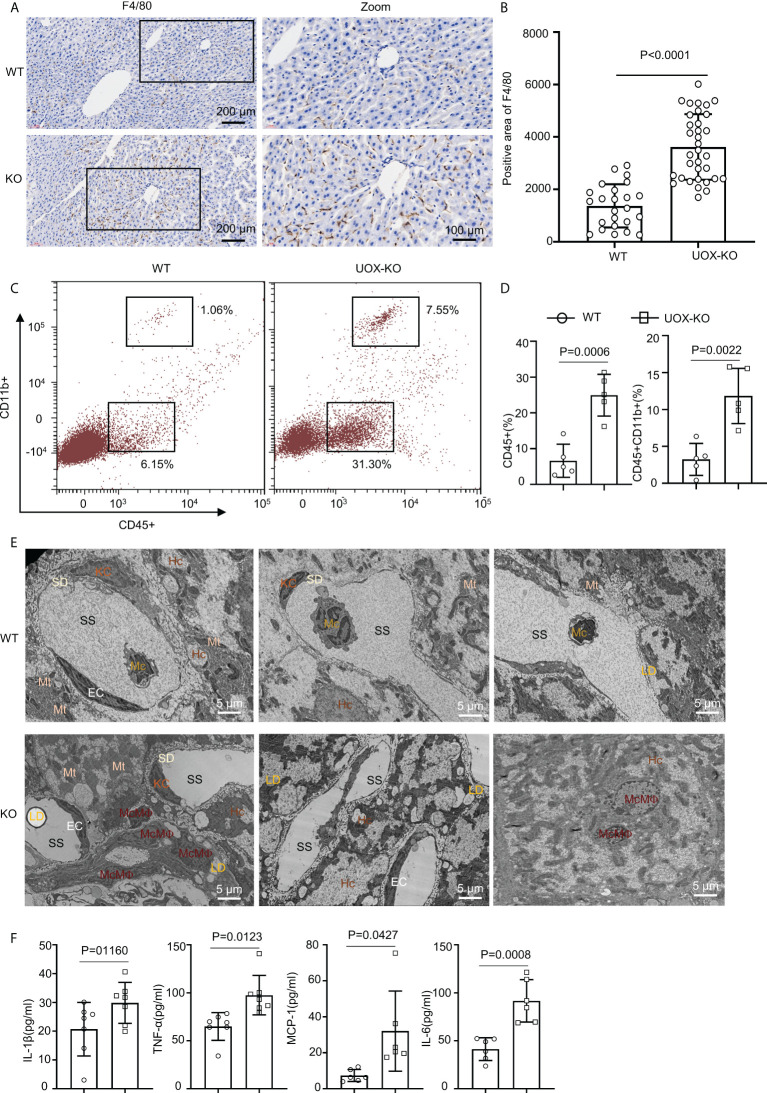
Liver from UOX-KO mice accumulated inflammatory macrophages. **(A)** Livers from 14-week-old WT C57BL/6J and UOX-KO mice underwent immunohistochemical staining with anti-F4/80 antibody to observe morphology; then, the sections were scanned using confocal scanning optical microscopy and quantified by ImageJ (six regions were selected for each mouse) **(B)**, scale bar = 200 or 100 μm. **(C, D)** The frequencies of total macrophages CD45^+^CD11b^+^ in the liver of WT and UOX-KO mice were analyzed and quantified by flow cytometry. **(E)** Transmission electron microscopy of WT or UOX-KO mice livers showing hepatocytes (Hc), Kupffer cells (KCs), monocyte-derived macrophages (McMΦs), monocytes (Mc), and Hc-associated subcellular structures such as the space of Disse or the perisinusoidal space (SD) and sinusoidal space (SS), scale bar = 5 μm. **(F)** The UOX-KO mice showed increased serum levels of proinflammatory cytokines. All the mice used in the experiment were male. The results are the mean ± SD (n = 6). Statistics: Student’s t-test. Differences were considered significant at P < 0.05.

Obesity conditions such as fatty liver involve an accumulation of inflammatory macrophages, with a key role in the pathogenesis of obesity-induced IR (18, 35–40). We assessed this possibility in the UOX-KO mice. The number of F4/80-positive cells increased in the UOX-KO livers compared to the WT mice (**Figures 3A, B**). We also analyzed the total CD45^+^CD11b^+^ macrophages in the liver tissue of the WT or UOX-KO mice by flow cytometry. In brief, the number of CD45^+^CD11b^+^ cells increased (**Figures 3C, D**), suggesting that these macrophages expressed a proinflammatory phenotype. Then, to determine the sources of F4/80^+^ cells in the liver of the UOX-KO mice, the hepatic cell content and structure were investigated by TEM (**Figure 3E**). Normal livers exhibited abundant hepatocytes and fewer KCs and hepatic stellate cells but few monocytes. UOX-mouse livers showed steatosis due to excess fat storage in the hepatocytes, which activated KCs to secrete a large variety of cytokines and chemokines, resulting in the recruitment of monocyte-derived macrophages (**Figure 3E**). The serum levels of IL-1β, IL-6, MCP-1, and TNF-α were all elevated in the UOX-KO mice (**Figure 3F**). Altogether, these results demonstrate that UOX-KO-mouse livers recruited inflammatory macrophages and showed increased circulating proinflammatory cytokines.

### HUA impaired glucose tolerance and insulin signaling in macrophages

**Figure 4:**
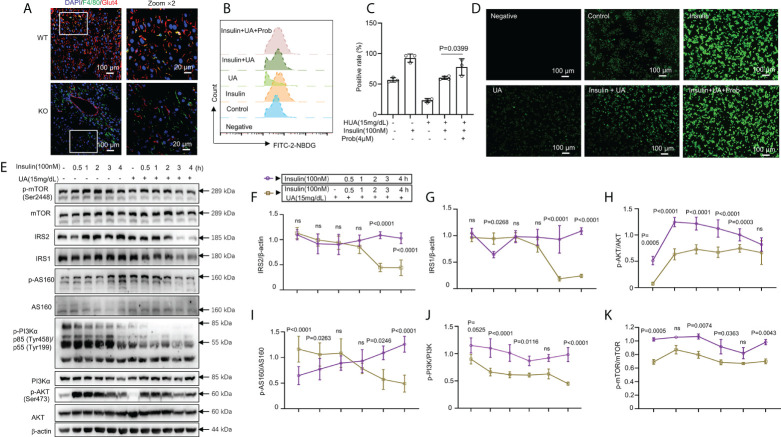
HUA impaired glucose tolerance and insulin signaling in macrophages. **(A)** Staining for F4/80 (a marker of macrophages, green) and glucose transporter 4 (GLUT-4, red) in frozen hepatic sections of 14-week-old WT mice and UOX-KO mice, scale bar = 100 or 20 μm. Bone marrow-derived macrophages (BMDMs) from WT mice were pretreated with HUA (15 mg/dL, 24 h) or probenecid (4 mM, 1 h), a UA transporter (OAT) inhibitor, and then underwent basal or insulin-stimulated 2-NBDG uptake assays detected by fluorescence microscopy **(B)** and analysis by flow cytometry **(C)**, scale bar = 100 μm. **(D)** Differentiated THP-1 cells were pretreated with UA (15 mg/dL) for 12 h, and insulin was added (100 nM). **(E–K)** Immunoblotting was used to analyze the phosphorylation of AKT, PI3K, and mTOR and the protein levels of AKT, IRS1, IRS2, PI3K, and mTOR. Data are the mean ± SD [**(A)**: n = 6, **(B-D)**: n = 3, **(E-K)**: n = 4]. Statistics: Two-way ANOVA with Tukey multiple comparisons tests. Differences were considered significant at P < 0.05. ns, No statistics.

Classically, GLUT4 is mainly responsible for skeletal muscle and adipocyte insulin-mediated glucose uptake (41–43). Previous studies have also confirmed that adipose-specific GLUT-4–KO mice showed IR secondarily in the muscle and liver tissue (42, 44). To explore glucose uptake in inflammatory macrophages in the liver of WT and UOX-KO mice, we assessed the co-localization of F4/80 and GLUT-4 by immunofluorescence assays. The UOX-KO mice exhibited the downregulation of GLUT-4 expression in hepatic macrophages, revealing an IR state in macrophages (**Figure 4A**). We then further explored the inherent glucose uptake of macrophages under HUA conditions. Insulin significantly increased 2-NBDG uptake, and pretreatment with 15 mg/dL of UA for 24 h suppressed insulin-induced 2-NBDG uptake (**Figures 4B–D**). Pretreatment with probenecid, a UA transporter (OAT) inhibitor, rescued HUA-decreased 2-NBDG uptake in macrophages (**Figures 4B–D**), suggesting that UA passed through the cell membrane to exert its functions, possibly *via* OAT channels.

The class-1 phosphatidylinositide 3-kinase (PI3K)-AKT pathway links the activation of the insulin receptor to glucose metabolism (45–50). Studies have confirmed that metabolic inflammation induces IR by inhibiting insulin signaling (51, 52). We evaluated the relationship between glucose uptake and insulin signaling. IRS1 and IRS2 levels were markedly reduced by combined treatment with UA (15 mg/dL, 24 h) and insulin (100 nM, 3 or 4 h) (**Figures 4E–G**). The phosphorylation of AKT, AS160, PI3K, and mammalian target of rapamycin (mTOR) was enhanced by insulin stimulation but reduced by treatment with 15 mg/dL of UA (**Figures 4E, H–K**). However, with prolonged insulin action, AKT, AS160, PI3K, and mTOR phosphorylation gradually decreased, which was sharply reversed by UA treatment (**Figures 4E, H–K**). Collectively, these observations indicate that insulin-driven PI3K-AKT signaling in macrophages was inhibited by UA treatment. Under hyperuricemic conditions, GLUT-4 was downregulated by inactivating insulin signaling.

### HUA mediated the IRS2 protein degradation pathway and activated AMPK/mTOR in macrophages

**Figure 5:**
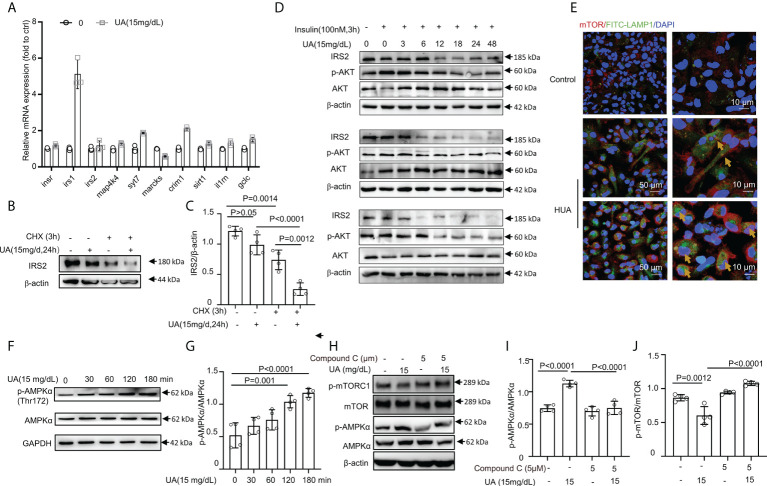
HUA mediated IRS2 protein degradation and activated AMPK/mTOR in macrophages. **(A)** BMDMs were exposed to HUA for 24 h, and the mRNA levels of genes associated with insulin signaling were assessed by RT-PCR. **(B, C)** Differentiated THP-1 cells were pretreated with UA; then, cycloheximide (CHX, 5 μM) was added for 3 h. IRS2 protein levels were evaluated by immunoblotting. **(D)** Differentiated THP-1 cells were pretreated with UA (15 mg/dL) for various periods; then, insulin (100 nM) was added for 3 h. Protein IRS2 and phosphorylated AKT levels were evaluated by immunoblotting and analyzed by ImageJ. Images are representatives of 3 independent experiments of biological replication. **(E)** Images of BMDMs incubated with UA (15 mg/dL, for 3 h) and co-immunostained with phalloidine and lysosomal-associated membrane proteins (LAMP1), showing the lysosomal localization of mTOR. Scale bar =10 μm. **(F, G)** Phorbol ester (PMA) (160 nm, for 24 h)-primed THP-1 cells were treated with UA (15 mg/dL) for various times, scale bar = 50 or 10 μm. Western blot analysis of total and phospho-AMPK levels. **(H–J)** THP-1 cells were pretreated with compound C for 2 h and then exposed to UA (15 mg/dL) for 2 h. HUA activated AMPK/mTOR pathway signaling in THP-1 cells, which was blocked by compound C, an inhibitor of AMPK. Data are the mean ± SD from 3–4 independent cell experiments. Statistics: One-way ANOVA with Tukey multiple comparison tests. Differences were considered significant at P < 0.05.

Recent data indicated that prolonged exposure to insulin could increase the degradation of IRS1 and IRS2 by the ubiquitin-proteasome system (53, 54). Such an increase in the proteasomal degradation of IRS1 is an attractive mechanism for IR because the increased ubiquitination of proteins is a common cellular mechanism for downregulating various signaling processes (55). We also found that IRS1 and IRS2 levels were decreased by combined treatment with UA (15 mg/dL, 24 h) and insulin (100 nM, 3 or 4 h) (**Figures 4E–G**). To further understand the mechanisms by which HUA inhibited insulin sensitivity, we assessed the IRS2 protein degradation pathway. The mRNA expression of genes associated with insulin signaling pathways, such as IR, IRS1, IRS2, MAP4K4, SYT7, and MARCKS, was assessed by RT-PCR. Except for IRS1 expression, the expression of the genes associated with insulin signaling did not change significantly (**Figure 5A**). However, IRS2 protein was degraded by combined treatment with CHX, a protein synthesis inhibitor, and UA (**Figures 5B, C**). Additionally, the IRS2 protein was degraded in a time-dependent manner by UA treatment, and the phosphorylated AKT level did not increase further (**Figure 5D**), suggesting that the IRS2 protein degradation pathway is involved in UA-induced IR.

Earlier studies showed that mTOR complex 1 (mTORC1) activation required mTORC1 localization to the lysosome, which is mediated by the formation of a complex between the active Rag heterodimer RagA/B-GTP/RagC/D-GDP and mTORC1 (Raptor and mTOR) (56, 57). We found that HUA induced the lysosomal localization of mTOR in BMDMs (**Figure 5E**). Since inflammation and IR are linked through the AMPK-mediated pathway, we assessed phosphorylated AMPK. Consistent with our hypothesis, AMPK was highly phosphorylated (**Figures 5F, G**), and mTOR activation was suppressed by UA treatment (**Figures 5H–J**). In addition, HUA activated AMPK/mTOR signaling in THP-1 cells, which was blocked by compound C, an inhibitor of AMPK (**Figures 5H–J**). Thus, UA inhibited the phosphorylation of mTOR and activated AMPK.

### IRS2 and its interacting protein-enriched ubiquitination degradation under HUA conditions

**Figure 6:**
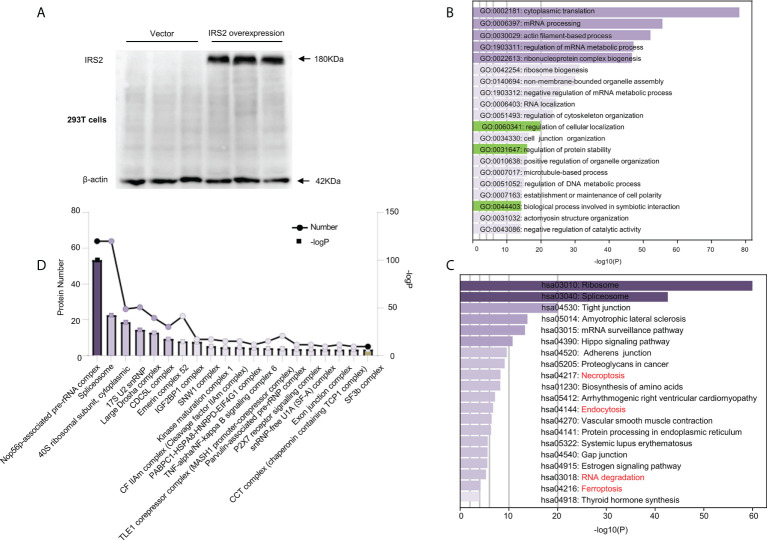
Outputs of proteomic analysis of IRS2-altered proteins under HUA conditions. Experimental workflow of the identification of IRS2-interacting proteins. HEK-293T cells were transduced with IRS2 with specific sequences containing the 3×Flag tag, followed by exposure to UA (15 mg/dL) for 24 h. The resulting cell lysates were reacted with Flag beads. **(A)** To confirm successful immunoprecipitation, IRS2 overexpression was identified using a Flag antibody. The groups were empty vectors and IRS2. To identify IRS2-binding proteins, the bound fraction was eluted, shotgun LC-MS/MS was performed, and the group set was as follows: IRS2 andUA+IRS2. **(B, C)** The pathways were analyzed for common genes using the Database for Annotation, Visualization, and Integrated Discovery (DAVID) ver. 6.8. GO analysis and KEGG pathway analysis represent the functional classes of IRS2-interacting proteins. **(D)** Proteins remarkably interacting with IRS2 were classified into different protein complexes. GO and KEGG analyses were performed using DAVID tools (https://david.ncifcrf.gov/). P < 0.01 was considered statistically significant.

Flag-IRS2 or empty vector was overexpressed in HEK-293T cells and immunoprecipitated using whole cell lysates in triplicate. HEK-293T cells expressing empty vector alone were used as the negative controls (**Figure 6A**). To identify the putative biological processes associated with IRS2-interacting proteins under HUA conditions, we used enrichment analysis with the GO domain “biological process” (**Figure 6B**) and KEGG pathway analysis (**Figure 6C**). These analyses identified the regulation of cellular localization and the regulation of protein stability, related to IRS2 degradation. Proteins significantly interacting with IRS2 were classified into different protein complexes. We identified 20 predominant complexes (**Figure 6D** and **Supplementary Dataset 2**. See https://doi.10.6084/m9.figshare.19450718): Nop56p-associated pre-rRNA complex, spliceosome, 40S ribosomal subunit, cytoplasmic, large drosha complex, CDC5L complex, emerin complex 52, IGF2BP1 complex, SNW1 complex, and other known complexes, as well as the SF3b complex. The autodegradation of E3 ubiquitin ligase COP1, the ubiquitin-dependent degradation of cyclin D, and the ubiquitin-mediated degradation of phosphorylated Cdc25A were also involved in IRS2-interacting proteins (**Supplementary Dataset 2**). Thus, ubiquitination degradation could be involved in IRS2 and its interacting proteins to contribute to IR under HUA conditions.

### Pharmacological proteasome inhibitor MG132 rescued insulin signaling and glucose uptake

**Figure 7:**
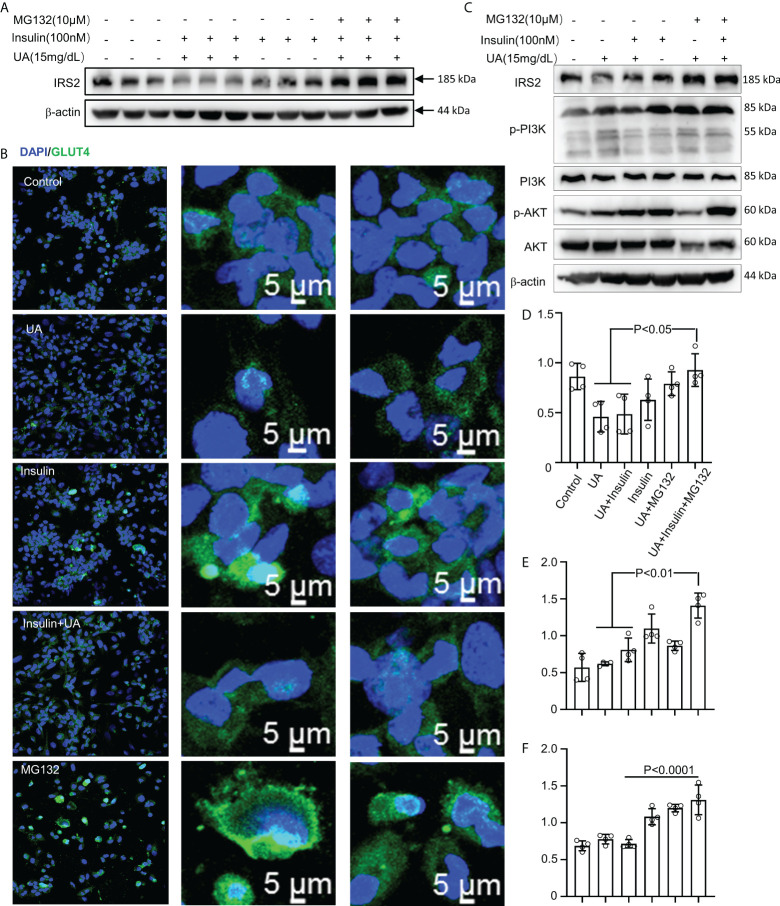
MG132 rescues insulin signaling and glucose uptake. **(A)** BMDMs were exposed to MG132 (10 μM) for 2 h before adding UA (15 mg/dL) for 24 h and insulin for 3 h. **(B)** GLUT-4 translocation was assessed by immunofluorescence staining. **(C)** Cells were pretreated with MG132 (10 μM) for 2 h, then exposed to UA (15 mg/dl) for 24 h, and insulin (100 nM) was added for 3 h. Immunoblotting was used to analyze the phosphorylation of AKT and PI3K(C). AKT, IRS2, and PI3K proteins were also quantified by immunoblotting **(D–F)**. Data are the mean ± SD from 4 independent cell experiments. Statistics: one-way ANOVA with Tukey's multiple comparison test. Differences were considered significant at P < 0.05.

HUA induced intrinsic IR in macrophages. Furthermore, treatment with the pharmacological proteasome inhibitor MG132 prevented the IRS2 degradation caused by combined stimulation with HUA and insulin (**Figure 7A**). To further investigate whether MG132 facilitated glucose uptake in HUA conditions, we assessed GLUT-4 translocation. Insulin increased GLUT-4 translocation to the cell surface in macrophages, which was significantly inhibited by HUA (**Figure 7B**). GLUT-4 translocation was increased significantly by MG132 pretreatment and combined stimulation with HUA and insulin (**Figure 7B**).

We next explored the effect of MG132 on insulin signaling in THP-1 cells exposed to UA (**Figure 7C**). HUA impaired IRS2/PI3K/AKT/AS160 signaling, which was rescued by treatment with MG132 (**Figures 7C-F**).

## Discussion

Our findings indicate that HUA-induced systematic IR may be associated with inflammation and the development of T2DM. UOX-KO mice showed metabolic disorders and systemic IR; inflammatory macrophages were recruited and circulating proinflammatory cytokine levels increased. We further demonstrated that hyperuricemia evoked glucose intolerance in liver macrophages, induced IR, and impaired insulin signaling. The fundamental cause of obesity is imbalanced food intake and energy expenditure, leading to chronic low-grade inflammation (58). These inflammatory responses inducing IR in obesity and T2DM are not limited to impaired insulin signaling pathways; complex interactions of multiple metabolic pathways have also been implicated (52, 59, 60). Of note, hyperuricemic mice did not show increased body mass but did show metabolic disturbances and IR, as evidenced by changes in glucose uptake, food and water consumed, oxygen consumption, the respiratory exchange ratio, and energy expenditure. In addition, the UOX-KO mice showed glucose intolerance and insulin insensitivity compared to the WT mice, as assessed by the GTT or the ITT. However, there was no difference in ^18^FDG uptake in the myocardium and muscle between the WT and UOX-KO mice, presumably due to the timing of PET/CT image acquisition. Whole-body PET images were acquired 30 min after the intravenous injection of ^18^F-FDG, and the ITT and GTT were monitored continuously for 120 min. Our previous studies demonstrated that HUA induced insulin resistance in skeletal muscle cells (61) and myocardial cells (7), suggesting that HUA-induced IR is associated with skeletal muscles and the myocardium.

Glycemic homeostasis is maintained by efficient communication between the insulin-secreting organ, the pancreas, and insulin target organs such as adipose tissue, the liver, and skeletal muscles in response to physiological challenges that transiently cause glycemia or lipemia. All tissues are filled with their own tissue macrophages, which maintain tissue homeostasis and physiological functions. IR is a breakdown of communication at insulin target tissues. At the onset of IR, macrophages accumulate in the adipose tissue (62), liver (63), and pancreatic islets (64). Since inflammation is linked to metabolic health, tissue macrophage responses are potent mediators of insulin signaling, sensitivity, and resistance but are often ignored. Compensatory hyperinsulinemia that ensues excessive dietary carbohydrate intake or early-stage insulin resistance in overweight or obese patients may provoke macrophages to release proinflammatory cytokines like IL-1β, which in turn may render insulin target cells insulin-resistant. Such a mechanism might be primarily relevant in the liver, which is exposed to much higher insulin concentrations than any peripheral organ (65). Kupffer cells contribute to the production of inflammatory mediators that promote insulin resistance in hepatocytes. Myeloid/macrophage-specific KO models such as IKK-β or CCR2 that impair inflammatory responses in Kupffer cells show attenuation of hepatic insulin resistance in the setting of high-fat feeding, despite the full development of hepatic steatosis (11, 66). This study showed that hyperuricemia-induced circulating monocytes were recruited into the mouse liver; these macrophages exhibited impaired glucose uptake, insulin sensitivity, and insulin signaling pathways. The hyperuricemia-induced metabolic disease may also involve functional integration between several organs *via* circulating factors released from mononuclear macrophages.

Next, we explored the mechanism of IR in macrophages. IRS2 was highly expressed in macrophages, and loss of IRS2 in myeloid cells improved glucose homeostasis and promoted resistance to high fat diet-induced metabolic dysfunction (67). However, a recent study reported that myeloid cell-specific Irs2-deficient mice exhibited impairment of IL-4-induced M2a-subtype macrophage activation as a result of the stabilization of the FoxO1/HDAC3/NCoR1 corepressor complex, resulting in IR under the high-fat diet condition (68). Many studies have also reported that IRS1 or IRS2 degradation is one of the causes of IR (55, 69). In this study, our data indicated that IRS2 proteasome degradation might contribute to IR in HUA conditions. Consistent with previous reports (70), phosphorylated AKT levels were also decreased, suggesting the impairment of the insulin pathway. However, long-term insulin exposure also degraded IRS1/IRS2. Macrophages exposed to UA alone could not degrade IRS2, but insulin stimulation for 3 h after UA treatment promoted IRS2 degradation. In addition, the pharmacological proteasome inhibitor MG132 restored insulin signaling and glucose uptake.

Ubiquitination plays a critical role in regulating insulin-like growth factor and insulin signaling (71–76). Several ubiquitin ligases, such as CUL7 (77), Cbl-b (78), SOCS1, SOCS3 (69), and ubiquitinate IRSs, promote their proteasomal degradation, inhibiting IGF and insulin signaling, which contributes to muscle atrophy, as well as IR. Additionally, we used IRS2 as bait to find IRS2-interacting proteins and found that various protein complexes were enriched, including E3 ubiquitin ligase COP1, cyclin D, and ubiquitin-mediated degradation of phosphorylated Cdc25A. COP1, an E3 ubiquitin ligase, is implicated in the ubiquitylation of various protein substrates to promote their proteasomal degradation, which increases p53 turnover by targeting it for proteasomal degradation (79). Thus, COP1 could act as a novel targeting IRS2 for ubiquitin-mediated degradation.

Recent findings indicated that the metabolic effects of AMPK might be mediated, at least in part, by the modulation of mTORC1 activity. We found that HUA activated AMPK/mTOR in macrophages. mTORC1 (ser2448) signaling has been implicated in insulin and nutrient and cellular energy status (80, 81), and mTORC1 is known to induce IRS1/2 serine phosphorylation, leading to the inhibition of IRS1/2 tyrosine phosphorylation and/or proteasomal degradation of IRS1/2 (82–84). AMPK is a well-known physiological inhibitor of the energy-consuming mTOR signaling pathway. Accordingly, the HUA-induced AMPK activation in this study may be a possible mechanism to explain the inhibition of mTORC1 activity. Therefore, we speculated that HUA promotes proteasomal degradation of IRS2 and may inhibit insulin signaling through mTORC1.

### Limitations of this study

Although HUA induced IR in HepG2 cells and hepatic tissue (8, 85), since hepatocytes are the most abundant cell type in the liver that responds to insulin, the effect of UA in insulin signaling was not evaluated in primary mouse hepatocytes. Although we demonstrated the mechanism of IR in macrophages, previous studies have attempted to define the reaction kinetics between IR and inflammation (51, 86). Some reported that local IR preceded inflammation, and others reported inflammation before IR (14). Under HUA conditions, we did not confirm a link between inflammation and IR. In terms of mechanism, although LC-MS analysis indicated that IRS2 was degraded by ubiquitin ligase, we did not further verify it in this study. In addition, crosstalk between hepatocytes and hepatic macrophages remains to be studied in the UOX-KO mice.

## Conclusion

In summary, the liver in hyperuricemic mice recruited inflammatory macrophages, which impaired glucose uptake. The binding of insulin to its receptor activated IRS1/2, which triggered downstream signaling cascades, including the phosphorylation of the PI3K/AKT/AS160 pathway, which regulated GLUT-4 translocation into the plasma membrane and promoted glucose uptake by macrophages (Graphical Abstract). In addition, HUA-mediated IRS2 protein degradation disrupted PI3K/AKT/AS160 signal transduction in macrophages. The pharmacological proteasome inhibitor MG132 restored insulin signaling and glucose uptake. Macrophage activation plays a critical role in hyperuricemia. Cellular targeting of Kupffer cells to undergo alternative activation might be an effective strategy for treating HUA-induced insulin resistance.

## Data availability statement

The datasets presented in this study can be found in online repositories. The name of the repository and accession number can be found below: https://figshare.com/s/90b6af1ad966dff6f244 and https://figshare.com/s/2e663d29bff39b0b4609.

## Ethics statement

The animal study was reviewed and approved by The Institutional Animal Care and Use Committee of Xiamen University.

## Author contributions

JC, HZ, and CZ contributed to the study of design. HZ, MW, JL, BC, CX, QW, YY, WY, DX, WL, FH, and CZ. performed experiments. HZ analyzed the data and wrote the manuscript. JC, TY, HK, WW, and CZ. contributed to the discussion and reviewed and edited the manuscript. All authors reviewed and edited the manuscript. JC and CZ are the guarantors of this work and, as such, have full access to all the data in the study and take responsibility for the integrity of the data and the accuracy of the data analysis. All authors contributed to the article and approved the submitted version.

## Funding

This work was supported by grants from the National Natural Science Foundation of China (81570772), the Natural Science Foundation of Guangdong Province (2015A030313434), the Natural Science Foundation of Fujian Province (2020J01018), and the Gout Research Foundation (Japan, 2019).

## Acknowledgments

The experiments were mainly carried out in the Center Laboratory, Xiang’an Hospital of Xiamen University. The authors would like to thank the Core Facility of Biomedical, Xiamen University, for assistance with the experimental apparatus and National-Local Joint Engineering Research Center of Entomoceutics, Dali University. The authors would also like to thank the Core Facility of Biomedical, Xiamen University (JingRu Huang, Xiang You, Haiping Zheng, Luming Yao, and Cixiong Zhang).

## Conflict of interest

The authors declare that the research was conducted in the absence of any commercial or financial relationships that could be construed as a potential conflict of interest.

## Publisher’s note

All claims expressed in this article are solely those of the authors and do not necessarily represent those of their affiliated organizations, or those of the publisher, the editors and the reviewers. Any product that may be evaluated in this article, or claim that may be made by its manufacturer, is not guaranteed or endorsed by the publisher.
