# Supplementary material for: Hyperuricemia contributes to glucose intolerance of hepatic inflammatory macrophages and impairs the insulin signaling pathway via IRS2-proteasome degradation
Source: Front Immunol. 2022 Sep 13;13:931087. doi: 10.3389/fimmu.2022.931087 (PMC9513153; doi:10.3389/fimmu.2022.931087)
Supplement: Supplementary file 1 [file DataSheet_1.pdf]

Supplementary Material

Hyperuricemia contributes to glucose intolerance of hepatic inflammatory macrophages and impairs the insulin signaling pathway via IRS2-proteasome degradation

Hairong Zhao[1,2], Jiaming Lu[1], Furong He[1], Mei Wang[2], Yunbo Yan[1], Binyang Chen[1], De Xie[1], Chenxi Xu[1], Qiang Wang[1], Weidong Liu[1], Wei Yu[1], Yuemei Xi[1], Linqian Yu[1], Tetsuya Yamamoto[3], Hidenori Koyama[3], Wei Wang[1], Chenggui Zhang[2*] and Jidong Cheng[1,3*]

Supplementary Methods

Pyruvate Tolerance Test

Hepatic gluconeogensis is estimated using pyruvate tolerance test that measures systemic elevation of glucose partly derived from pyruvate and hepatic gluconeogensis following an intraperitoneal bolus injection of pyruvate in awake mice. In detail, WT mice (n=3, at 8 weeks) and UOX-KO mice (n=2, at 8weeks) were fasted overnight (~15 hours) prior to the start of experiment. Collect plasma sample (10 µl) before the start of experiment (basal-0 min) to measure basal glucose levels. Administer intraperitoneal injection of pyruvate (2 g/kg body weight, P5280, Sigma) using an insulin syringe. Collect plasma samples (10 µl) at 10, 20, 30, 45, 60, 90, and 120 min following pyruvate injection to measure circulating glucose concentrations.For data analysis, plasma glucose levels vs. time after pyruvate injection are plotted, and area-under-curve may be calculated to estimate hepatic gluconeogenesis.

Oil Red O Staining

To observe hepatic steatosis in mice, frozen serial cross-sections of liver tissue were stained with Oil red O (Cat# C0158S, Beyotime, China) (1).

Supplementary Figures

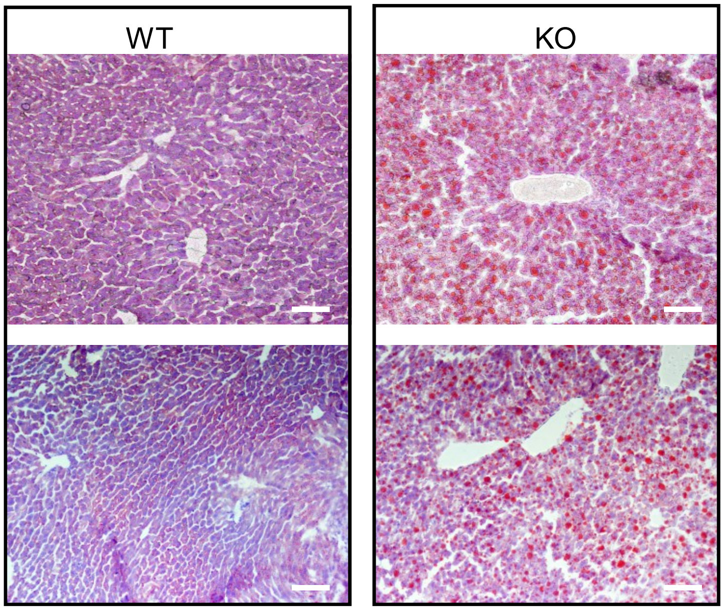

Supplementary Fig.1 Hepatic steatosis in UOX-KO mice must be confirmed by Oil Red O staining.

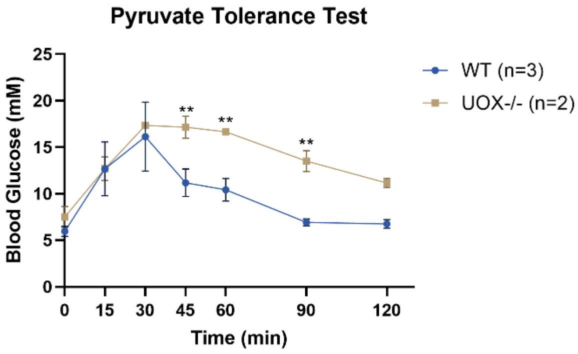

Supplementary Fig.2 Pyruvate tolerance test.

1. Xie, Zhao H, Lu J, He F, Liu W, Yu W, Wang Q, Hisatome I, Yamamoto T, Koyama H, and Cheng J, High uric acid induces liver fat accumulation via ROS/JNK/AP-1 signaling. *Am J Physiol Endocrinol Metab*(2021) 320(6):E1032-E43.doi: 10.1152/ajpendo.00518.2020.
